# Spatial contextual cues that help predict how a target will accelerate can be used to guide interception

**DOI:** 10.1167/jov.23.12.7

**Published:** 2023-10-23

**Authors:** Emily M. Crowe, Jeroen B. J. Smeets, Eli Brenner

**Affiliations:** 1 Department of Human Movement Sciences, Institute of Brain and Behavior Amsterdam, Amsterdam Movement Sciences, Vrije Universiteit Amsterdam, Amsterdam, The Netherlands; 2 School of Psychology, University of Nottingham, University Park, United Kingdom; 3 Department of Human Movement Sciences, Institute of Brain and Behavior Amsterdam, Amsterdam Movement Sciences, Vrije Universiteit Amsterdam, Amsterdam, The Netherlands; 4 Department of Human Movement Sciences, Institute of Brain and Behavior Amsterdam, Amsterdam Movement Sciences, Vrije Universiteit Amsterdam, Amsterdam, The Netherlands

**Keywords:** goal-directed movement, error compensation, rolling wheel

## Abstract

Objects in one's environment do not always move at a constant velocity but often accelerate or decelerate. People are very poor at visually judging acceleration and normally make systematic errors when trying to intercept accelerating objects. If the acceleration is perpendicular to the direction of motion, it gives rise to a curved path. Can spatial contextual cues help one predict such accelerations and thereby help interception? To answer this question, we asked participants to hit a target that moved as if it were attached to a rolling disk, like a valve (target) on a bicycle wheel (disk) moves when cycling: constantly accelerating toward the wheel's center. On half the trials, the disk was visible such that participants could use the spatial relations between the target and the rolling disk to guide their interception. On the other half, the disk was not visible, so participants had no help in predicting the target's complicated pattern of accelerations and decelerations. Importantly, the target's path was the same in both cases. Participants hit more targets when the disk was visible than when it was invisible, even when using a strategy that can compensate for neglecting acceleration. We conclude that spatial contextual cues that help predict the target's accelerations can help intercept it.

## Introduction

Objects in the environment do not always move at a constant velocity. An approaching tennis ball, for example, will accelerate and decelerate as a result of gravity, of hitting the ground, and of any spin that the opponent used. Despite acceleration being an essential feature of most moving objects, people are very poor at visually judging it (e.g., Brenner et al., 2016; Benguigui, Ripoll, & Broderick, 2003; Brouwer, Brenner, & Smeets, 2002; Calderone & Kaiser, 1989; Gottsdanker, Frick, & Lockard, 1961; Merfeld, Zupan, & Peterka, 1999; Mueller, González, McNorgan, Steinbach, & Timney, 2017; Watamaniuk & Heinen, 2003; Werkhoven, Snippe, & Tote, 1992). It is therefore not surprising that people make systematic errors when trying to intercept accelerating targets (e.g., Brouwer et al., 2002; Kreyenmeier, Kämmer, Fooken, & Spering, 2022; Port, Lee, Dassonville, & Georgopoulos, 1997). They do so because they cannot reliably consider acceleration when predicting how the target's trajectory will proceed (e.g., Benguigui & Bennett, 2010; Dubrowski & Carnahan, 2002). The systematic errors correspond with people constantly updating the judged velocity of the target without considering the acceleration: They underestimate the displacement of accelerating targets and overestimate that of decelerating ones (Brenner & Smeets, 2015).

Interception errors decrease through practice if there is regularity in the acceleration over repeated trials. This might suggest that people learn to anticipate certain accelerations of the target and control the timing of their interception accordingly (e.g., de Rugy, Marinovic, & Wallis, 2012; Diaz, Cooper, Rothkopf, & Hayhoe, 2013; Fialho & Tresilian, 2017; Mann, Nakamoto, Logt, Sikkink, & Brenner, 2019; Zago, McIntyre, Senot, & Lacquaniti, 2009). However, the improvement in performance could simply result from people adjusting their movements to feedback: compensating for the error on the previous movement rather than learning to anticipate a certain acceleration (Brenner, de la Malla, & Smeets, 2023). When participants intercepted targets moving across images of surfaces that normally have different friction coefficients, and that indeed indicated how quickly the targets would decelerate, they continued to make systematic errors in line with those seen when no contextual information about the friction coefficient was provided (Brenner et al., 2016). In contrast, when participants intercepted targets with different decelerations in different blocks, their performance improved. Thus, participants adjusted their movements in response to feedback even when contextual information indicated how the target would decelerate (Brenner et al., 2016). Such contextual information requires consideration of the depicted surface. Does providing more direct spatial contextual information that helps participants predict the changing accelerations of the target they are moving to intercept help performance?

A ventral visual pathway that connects the primary visual cortex with the inferior temporal cortex processes information that is needed for recognizing objects. A dorsal visual pathway running from the primary visual cortex to the posterior parietal cortex processes information that is needed to guide ongoing actions. The influential two visual streams hypothesis proposes that these two cortical pathways use different visual reference frames (Goodale & Milner, 1992). The ventral pathway is assumed to use persistent object properties such as the relative positions of structures within the object (i.e., in an allocentric reference frame so that the position of the observer is irrelevant) to support tasks such as object recognition (Goodale, 2008). The dorsal pathway is assumed to use instantaneous information about the object's position relative to the observer (i.e., the object's position in an egocentric reference frame) to guide movements. According to this strict functional segregation, providing spatial contextual cues should not improve interception of an accelerating target, because such interception relies on updating the real-time, egocentric position of the target relative to oneself.

More recent evidence argues against such a strict functional segregation and suggests that allocentric and egocentric reference frames are used by both the dorsal and ventral stream (see Schenk & McIntosh, 2009, for a review). It is thus conceivable that providing spatial contextual information in the form of a fixed spatial relationship between structures could aid interceptive performance. This fits with the notion that the involvement of the two visual streams is determined by the visual attribute that is used, rather than by whether the task being performed is an action or a perceptual judgment (de la Malla, Brenner, de Haan, & Smeets, 2019; Smeets, Brenner, de Grave, & Cuijpers, 2002).

In this experiment, we examined whether interception performance improves when one provides spatial contextual information that helps predict a target's accelerations and the resulting complicated trajectory. We asked participants to hit a target that moved with a disk rolling down a slope, similarly to how a valve moves on a bicycle wheel as the wheel rolls along its path. On half the trials, the disk was visible such that participants could use the spatial relation between the target and disk to see why the target was accelerating or decelerating. On the other half of the trials, the disk was not visible. Importantly, the target always followed the same path. According to the functional segregation proposed by the two visual streams hypothesis (Goodale & Milner, 1992), providing spatial contextual information should not benefit performance in interceptive actions. Thus, participants should perform similarly regardless of whether the disk is visible or not. Alternatively, since the spatial contextual information can help participants predict the target's motion and is thus a relevant visual attribute in guiding the movement (de la Malla et al., 2019; Smeets, Brenner, de Grave, & Cuijpers, 2002), we expect to see better performance when the disk is visible than when it is invisible.

One way to deal with having to predict how a target in our experiment will accelerate is to use a *constant-phase strategy.* For a target attached to a rolling disk, the direction of the target's instantaneous acceleration is determined by the phase of the rotation. As already mentioned, if targets accelerate in the same way across multiple trials, people learn to compensate for the systematic errors that arise from ignoring the acceleration by simply aiming further to one side (Brenner et al., 2016; Brenner et al., 2023). If one tries to hit the target when it reaches the same phase on every trial, neglecting the target's acceleration will always lead to the same error, so correcting for the error on the next trial will be effective. We therefore expect participants who consistently hit the target when it reaches the same phase of the motion to hit more targets.

A way to improve interception without compensating for acceleration at all is a *low-velocity* strategy. This strategy exploits the fact that the precision of interception is better for slower targets because any timing error matters less if the velocity is low (Brouwer, Brenner, & Smeets, 2000). In our rolling disk paradigm, the target reaches its lowest velocity when it is closest to the surface. The low-velocity strategy is thus a very specific realization of the constant-phase strategy.

We will investigate whether providing spatial contextual cues in the form of showing the rolling disk to participants improves performance and whether this improvement is accompanied by the reduction of systematic errors that one would expect if participants are able to predict the acceleration. Furthermore, we will investigate whether improvement of performance is related to the use of a constant-phase or a low-velocity strategy.

## Methods

### Participants

Fifty-one participants took part in the experiment (28 females; 49 right-handed; 26 ± 6 years old). Participants either volunteered to take part or took part in return for course credit. The study was approved by the local ethics committee in accordance with the Declaration of Helsinki.

### Setup

The experiment was conducted in a normally illuminated room. The stimuli were back-projected at 120 Hz with a resolution of 800 × 600 pixels onto a 1.25-m × 1.0-m acrylic rear-projection screen (Techplex 15; Stewart Filmscreen Corporation) tilted backward by 30°. An infrared camera (Optotrak 3020; Northern Digital) that was placed at about shoulder height to the left of the screen measured the position of a marker (an infrared light-emitting diode) attached to the nail of the index finger of the participant's dominant hand at 500 Hz.

In order to synchronize the timing of the movement data (i.e., the marker position) with the stimulus presentation, the camera also recorded the position of a second marker attached to the side of the screen. This marker did not move, but it stopped emitting infrared light so that its position was registered as “missing” when a flash was presented at the top left corner of the screen (where a light sensor was placed to detect the flash). We used a simple 4-point calibration to relate the position of the fingertip to the projected images, automatically correcting for the fact that the marker was attached to the nail rather than the tip of the finger.

### Stimulus and procedure

Participants stood in front of a large screen and were free to move as they wished. They were instructed to try to intercept the black target (a 2-cm diameter dot) and were free to hit it at any point before it left the screen. To start a trial, participants placed their finger within the green starting point (a 3-cm diameter dot) that was presented 28 cm below the screen center. Between 600 and 1,200 ms after they did so, the target appeared in the top-left corner of the screen. The black target was attached to a white disk (20 cm diameter) that rolled down a blue slope. On half of the trials, these items were presented on a gray background, so the disk was clearly visible. On the other half of the trials, the background was white, such that the white disk was not visible, and the target disc appeared in isolation. We chose to vary the background color rather than that of the disk so that the contrast at the edge of the target was always the same. The starting position of the center of the disk was always 40 cm left and 19 cm above the screen center, at the top of a blue downward slope (2-cm × 100-cm rectangle, oriented at –10 degrees). The disk rolled down the slope at a constant velocity of 61 cm/s. The center of the target was attached to the disk at 7.5 cm from its center, such that it followed a curtate trochoid. The initial orientation of the disk was chosen at random on each trial, so that the target's trajectory varied across trials so intercepting the target at the same phase does not correspond with intercepting it at the same position. Participants received feedback if they correctly intercepted the target (i.e., the calibrated finger position was within the target when they tapped the screen): They heard a sound and the target remained at that position for 500 ms. If participants missed the target, they heard nothing, and the target deflected away from their finger at 100 cm/s. Participants chose when to begin the next trial by placing their finger in the starting point such that it was a self-paced task. Figure 1 shows how the target moves in the two different conditions.

**Figure 1.** Illustration of the target's motion in the two conditions: disk invisible (left) or visible (right). The black dot is the target. It is attached to a white disk rolling down the blue slope. The example is a single trial that is looped. Each panel shows the target's path (top; see Johansson, 1973) and its velocity (middle) and direction of motion (bottom) as a function of its horizontal position (how this relates to time can be judged from the spacing between the dots along the target's path that are equally spaced in time). The mean direction is below zero (horizontal lines) because the disk is rolling down the slope. Movie is available on the journal website.

### Design

The experiment used a within-subject design with one independent variable: disk visible or invisible. Each participant completed one block of 400 trials (200 trials in each condition: disk visible or invisible) in a single session that took approximately 20 min. The two conditions were randomly interleaved. We were interested in three dependent variables: the fraction of targets hit, the phase of the target's motion when the screen was tapped, and the position of the tap with respect to the target.

## Results

**Figure 2.**
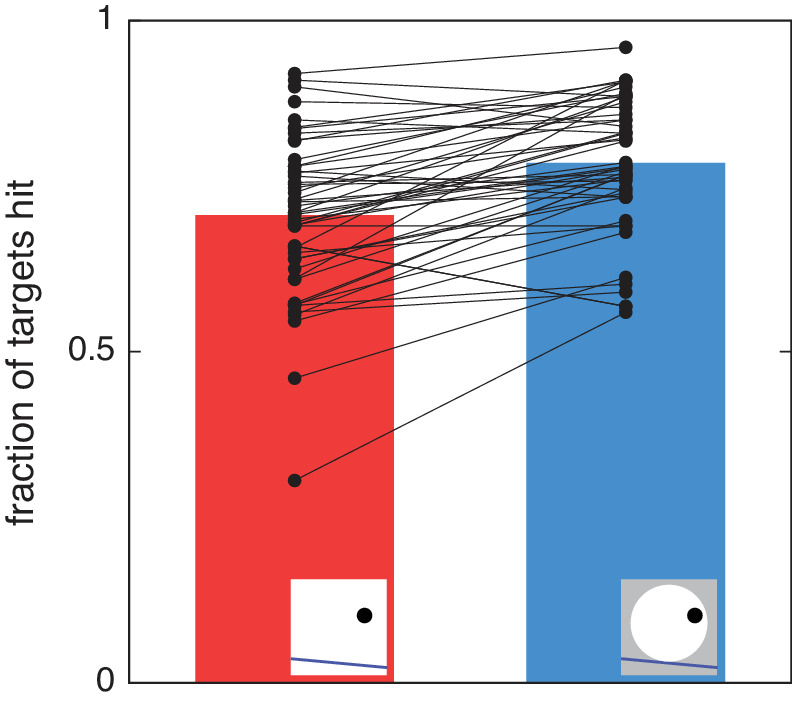
Fraction of targets hit when the disk was invisible (red) and when the disk was visible (blue). The data of individual participants are shown by black points connected by a line. Almost all participants hit more targets when the disk was visible.

Participants hit more targets when the disk was visible (blue bar in Figure 2) than when it was invisible (red bar in Figure 2; Wilcoxon signed-rank test, *T* = 107, *p* < 0.0001). This shows that having spatial contextual cues aids interception performance. The time between the target's appearance and it being tapped (mean ± standard error) was similar in both the disk-invisible (772 ± 25 ms) and disk-visible (763 ± 23 ms) conditions.

**Figure 3.**
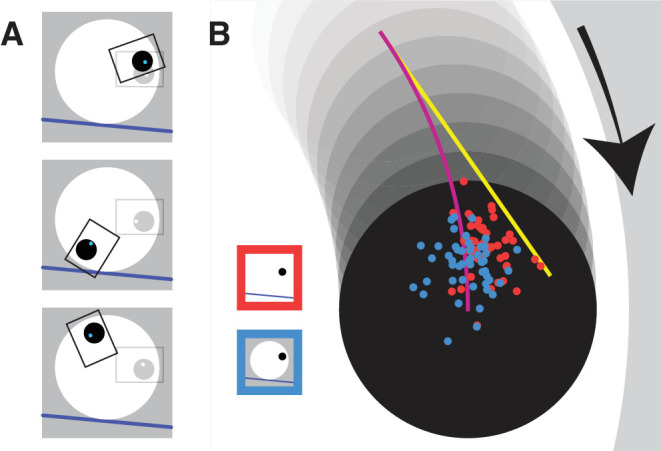
(**A**) Three examples of the phase-alignment procedure. The tapping position on a given trial is indicated by a blue dot within the black target presented at its location at the time of the tap. This tap and target position on every trial were rotated to align the areas indicated by the black rectangles in the orientation indicated by the gray rectangles to allow averaging across trials. (**B**) Mean tapping locations of individual participants when the disk was invisible (red dots) or visible (blue dots). The position of the target at the time of the tap is shown in black, with its previously occupied positions during 100 ms shown in shades of gray. The yellow line shows how the target center would have moved if it had continued to move in the same direction from 100 ms before the tap. The purple curve shows how the target center moved during the last 100 ms before the tap. Any systematic difference between the red and blue tapping points indicates that visibility of the disk influences where participants aim.

To investigate our proposal that the spatial contextual information available in the visible-disk condition helps predict the target's accelerations, we investigated *where* participants tapped on the screen with respect to the moving target. To do so, we determined the mean tapping error across all trials for each participant in both conditions (Figure 3). If participants ignore the target's acceleration during the 100-ms visuomotor delay (Brenner & Smeets, 2015), they will aim at the end of the yellow line in Figure 3: a bit behind the center of the target and toward the “outside” of the disk. We expect this to happen when the disk is not visible. If participants use the fact that they can see the white disk to correctly anticipate that the target will accelerate toward the center of the disk, they will aim at the target center (the end of the purple curve in Figure 3). Figure 3 shows that many participants used the visible disk to predict the target's acceleration: The blue dots cluster closer to the purple line than the red ones. Note that the red dots occupy the space between the yellow and purple lines, showing that participants did predict some target acceleration when the disk was invisible.

**Figure 4.**
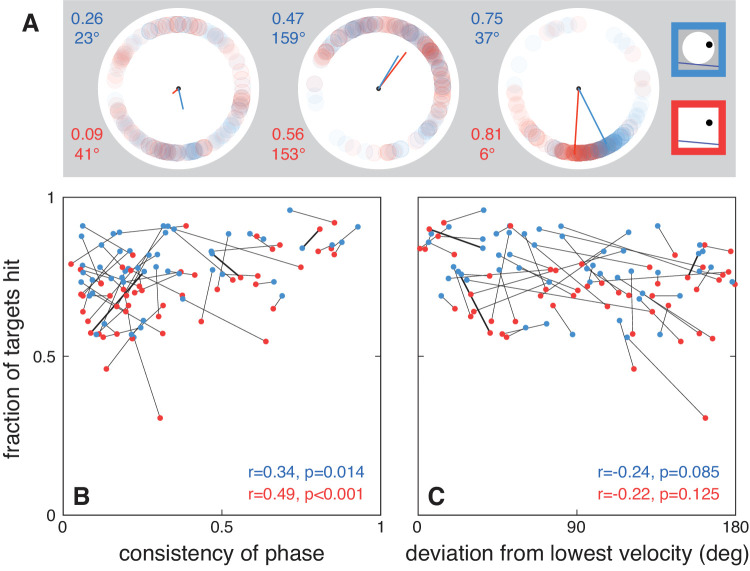
Possible interception strategies. (**A**) The positions of the target with respect to the center of the disk (phase) at the time of the tap for three participants. The lines show the vector averages of the corresponding positions, so that their lengths indicate the consistency of phase. From left to right, these participants showed a low, middle, and high consistency of phase. The direction of the line was used to determine the deviation of the average direction from that at which the target has its lowest velocity. The participant whose data are shown in the central panel usually tapped when the target was moving fast while the participant whose data are shown on the right usually tapped when the target's velocity was low. The lines connecting the data from these three participants are presented in bold in B and C. The numbers are the lengths and orientations of the lines. (**B**) The consistent phase strategy: The fraction of targets hit increases with the consistency of the phase in the target's motion at the time of the tap. Each participant is represented by a pair of dots connected by a line. The numbers show the correlation coefficient and the *p* value associated with a comparison with there being no correlation. (**C**) The low-velocity strategy: The fraction of targets hit tends to decrease with the deviation of the average phase from the phase at which the target's velocity was at its lowest.

To determine whether participants used a constant-phase strategy and whether doing so improved performance, we looked at the consistency in the target's rotational phase when the screen was hit (see examples in Figure 4A) and whether this consistency was related to the fraction of targets hit (Figure 4B). The consistency varied considerably between participants. Participants who generally tapped on the screen when the target was in the same phase of its rotational movement (consistency values close to 1 in Figure 4B), rather than, for instance, at a fixed time after the target appeared, hit more targets. This was the case both when the disk was visible and when it was invisible, although it was less beneficial when the disk was visible (blue dots in Figure 4B). This is presumably a ceiling effect, because the spatial contextual cues also helped participants to predict the target's accelerations in the visible-disk condition.

The rightmost example participant in Figure 4A consistently tapped on the screen when the target was close to the surface, which was when its speed was at its lowest. To check whether the observed benefit of the constant-phase strategy is the consequence of participants tapping when the target's speed was at its lowest (the low-velocity strategy), we also looked at the relationship between the fraction of targets hit and the target's most likely velocity at the time of the tap. This correlation was not statistically significant (Figure 4C). The advantage of tapping when the target reaches a certain phase is therefore not just a side effect of participants selecting the phase in which the target is moving at its slowest. Although the best performance does appear to be achieved when using the low-velocity strategy, it is evident from Figure 4C that few participants actually consistently used this strategy or even the constant-phase strategy (Figure 4B).

## Discussion

This experiment shows that providing spatial contextual cues can help participants intercept accelerating targets: Participants hit more targets when the rolling wheel that the target was mounted on was visible than when it was invisible (Figure 2). This finding fits with the idea that goal-directed movements use visual attributes that are relevant for the task (de la Malla et al., 2019; Smeets et al., 2002). Specifically, the visible wheel helped participants predict the target's accelerations, thus providing helpful information about the target's motion to facilitate interception. This finding adds to the growing body of evidence against a strict functional segregation between visual pathways for perception and action (Medendorp, de Brouwer, & Smeets, 2018; Schenk & McIntosh, 2009): Providing a spatial contextual cue improved interception despite the same real-time egocentric information about the target being available to participants in the two conditions.

Even when the wheel was invisible, participants compensated for some of the acceleration of the target (red dots in Figure 3 are not centered around the yellow line but biased toward the purple curve). How did they do this? People can visually judge acceleration to some extent (Benguigui et al., 2003; Brouwer et al., 2002; Watamaniuk & Heinen, 2003), so maybe what we observe is the result of relying on such information. They might also recognize regularities in the target's motion and use this to guide their movements (de Rugy et al., 2012; Diaz et al., 2013; Fiahlo & Tresilian, 2017; Mann et al., 2019; Zago et al., 2009). It is evident that part of the apparent consideration of acceleration is the result of correcting for earlier errors (Brenner et al., 2023), because participants performed better if they consistently hit the target at the same phase (Figure 4B). The consequence of doing so is that the target is accelerating in the same direction just before the hit, so that compensating for the error on the next trial reduces the error on that trial (Brenner et al., 2016; Brenner et al., 2023). This benefit was particularly evident in the invisible wheel condition, presumably because participants did not have the spatial contextual cues to help them predict the target's acceleration.

Since the precision of interception is better for slower targets, because any timing errors matter less when the velocity is low (Brouwer et al., 2000), one might expect participants to use a low-velocity strategy, whereby they aim at a position where the target moves slowly (i.e., when it is close to the surface). This strategy would also lead to participants consistently hitting the target in its same rotational phase. To check whether the advantage of a constant-phase strategy was only a consequence of participants aiming to hit the target at its lowest velocity, we also looked at the relationship between the fraction of targets hit and the deviation from the target's lowest velocity. This relationship was not significant in either condition (Figure 4C), in contrast to the correlation between performance and the consistency in phase. Indeed, several participants who were quite successful were consistent in the phase of the target at the time of the tap but intercepted the target when it was close to its highest velocity (the participant of the middle panel of Figure 4A is one of them; this participant is indicated by the central thick line in Figure 4B and the rightmost thick line in Figure 4C). So, the advantage of using a constant phase is not simply the consequence of using a low-velocity strategy.

Spatial contextual cues improve interception performance by helping participants predict the complicated accelerations and decelerations of the target. In addition, participants who consistently hit the target at the same phase of its rotational movement hit more targets, presumably because it allowed them to use their errors on previous trials to adjust their movement. We conclude that goal-directed movements use any visual information that is relevant for the task and can be judged well enough to be useful.
